# Synergistic Effect of Various Virulence Factors Leading to High Toxicity of Environmental *V. cholerae* Non-O1/ Non-O139 Isolates Lacking *ctx* Gene : Comparative Study with Clinical Strains

**DOI:** 10.1371/journal.pone.0076200

**Published:** 2013-09-23

**Authors:** Neha Rajpara, Kittappa Vinothkumar, Priyabrata Mohanty, Arun Kumar Singh, Rajesh Singh, Ritam Sinha, Dhrubajyoti Nag, Hemanta Koley, Ashima Kushwaha Bhardwaj

**Affiliations:** 1 Department of Human Health and Diseases, School of Biological Sciences and Biotechnology, Indian Institute of Advanced Research, Gandhinagar, Gujarat, India; 2 Department of Cell Biology, School of Biological Sciences and Biotechnology, Indian Institute of Advanced Research, Gandhinagar, Gujarat, India; 3 National Institute of Cholera and Enteric Diseases, Beliaghata, Kolkata, India; University of Osnabrueck, Germany

## Abstract

**Background:**

*Vibrio cholerae* non-O1/ non-O139 serogroups have been reported to cause sporadic diarrhoea in humans. Cholera toxins have been mostly implicated for hypersecretion of ions and water into the small intestine. Though most of the *V. cholerae* non-O1/ non-O139 strains lack these cholera toxins, several other innate virulence factors contribute towards their pathogenicity. The environmental isolates may thus act as reservoirs for potential spreading of these virulence genes in the natural environment which may cause the emergence of epidemic-causing organisms.

**Results:**

The environmental isolates of *vibrios* were obtained from water samples, zooplanktons and phytoplanktons, from a village pond in Gandhinagar, Gujarat, India. They were confirmed as *Vibrio cholerae* non-O1/ non-O139 using standard biochemical and serotyping tests. PCR experiments revealed that the isolates lacked *ctxA, ctxB, tcpA, zot* and *ace* genes whereas other pathogenicity genes like *toxR*, *rtxC*, *hlyA*, *hapA* and *prtV* were detected in these isolates. Compared with epidemic strain *V. cholerae* O1 El Tor N16961, culture supernatants from most of these isolates caused higher cytotoxicity to HT29 cells and higher hemolytic, hemagglutinin and protease activities. In rabbit ileal loop assays, the environmental isolates showed only 2-4 folds lesser fluid accumulation in comparison to N16961 and a *V. cholerae* clinical isolate IDH02365 of 2009. Pulsed Field Gel electrophoresis and Random amplification of Polymorphic DNA indicated that these isolates showed considerable diversity and did not share the same clonal lineage even though they were derived from the same water source. All the isolates showed resistance to one or more antibiotics.

**Conclusion:**

Though these environmental isolates lacked the cholera toxins, they seem to have adopted other survival strategies by optimally utilising a diverse array of several other toxins. The current findings indicate the possibility that these isolates could cause some gastroenteric inflammation when ingested and may serve as progenitors for overt disease-causing organisms.

## Introduction

*Vibrio cholerae* has been associated with severe cases of diarrhoea with high case fatality rate. More than 200 serogroups of *V. cholerae* are known, out of which only two, O1 and O139 serogroups cause epidemics or outbreaks. Non-O1/ non-O139 serogroups of *V. cholerae* have also been associated with sporadic cases of diarrhoea and have been implicated as progenitors of new variants of epidemic *V. cholerae* strains [1,2]. A few pathogenic strains of non-O1 and non-O139 like O141, O10 and O12 have caused outbreaks of gastroenteritis though they lack ctx toxin [3-5]. It was then proposed that these strains caused the disease by a mechanism different from that of toxigenic *V. cholerae* and were termed as enteropathogenic *V. cholerae* [6-9]. Pathogenic as well as non-pathogenic non-O1/non-O139 *V. cholerae* are found in aquatic ecosystems but non-pathogenic strains typically dominate this niche. The mechanism by which cholera becomes endemic depends on the environmental reservoir of *V. cholerae*. Major outbreaks of cholera usually result from an interplay of factors such as favourable climate conditions and poor sanitation. Cholera pathogenesis is a cumulative effect of a number of virulence factors. Studies have implicated toxin-coregulated pilus (TCP), cholera toxin (CT) and toxin regulatory protein (Tox R) which coregulates the expression of CT and TCP, as the major virulence factors for cholera in humans [10]. Lots of studies have shown that the other proteins like mannose-fucose-resistant cell-associated hemagglutinin, mannose-sensitive hemagglutinin and outer membrane proteins play a role in enhancing the adhesion and intestinal colonization [10]. Though the virulence factors for the non-O1/non-O139 strains are inadequately understood, there are reports implicating a hemagglutinin protease, few other proteases and toxins in the pathogenicity of these strains [6,11-14]. A microarray-based analysis of four non-O1/non-O139 strains implicated a type three secretion system in the virulence and the environmental fitness of these strains [8]. Microorganisms produce various extracellular and intracellular proteases to invade host defense system and modulate colonization. *V. cholerae* have various levels of arsenal for colonization and infection. Primary line is controlled by *ctx* genes. In absence of these genes other accessory toxic genes like hemagglutinin protease (HAP), *V. cholerae* protease (PrtV) and a serine protease (VC1649) help to modulate the infection in the small intestine [11,14]. HAP is a versatile metalloprotease that performs several functions like increased inflammation in small intestine, disruption of cell to cell junctions resulting in increased hemorrhagic fluid loss and detachment of *V. cholerae* from epithelial cells [11,14]. PrtV proteases have been shown to increase survival of *V. cholerae* in environmental niches. Comparative genomics studies have demonstrated that environmental *Vibrios* lack toxin-encoding genes in their chromosomes but may capture the virulence genes through different mobile genetic elements and become toxic [15]. Thus, environmental strains of *Vibrio* spp. may act as reservoirs for potential spreading of virulence genes in the natural environment [16,17]. The ligated rabbit ileal loop assay has been used routinely to assess the secretory responses from diarrhoea producing bacterial strains and the environmental strains have also been evaluated for their enterotoxigenicity using this assay [18-20].

Therefore, monitoring of non-O1/non-O139 environmental strains could help in strengthening the cholera surveillance in any location. Though there have been no reports of outbreaks of cholera from Gandhinagar region, Gujarat state, the study was initiated to understand the toxic potential of the environmental isolates. Our results have revealed that these strains elaborate some extracellular toxins having protease, hemagglutination and hemolytic potential and could serve as the progenitors for the toxigenic *V. cholerae* under favourable environmental conditions.

## Methods

### Isolation of environmental samples, bacteriology and serogrouping

Nine *Vibrio cholerae* isolates were obtained from zooplankton, phytoplankton and water samples from a pond of Lawarpur village, Gandhinagar, Gujarat in 2009, as per the published protocols [21-24]. Water from this pond is used by the villagers for various purposes like washing and bathing. All these samples were first enriched in Alkaline Peptone Water (APW) medium and then selected on Thiosulfate Citrate Bile Sucrose (TCBS) agar (Difco Laboratories, Detroit, Michigan). Three yellow, sucrose-fermenting colonies with elevated centers were chosen from each type of sample and characterized by a series of biochemical tests for identification [25]. The serogroup analysis was done using *V. cholerae* antisera (Denka Seiken Co., Japan). The isolates were named as Z1, Z2, Z3 (derived from zooplankton); P1, P2, P3 (derived from phytoplankton) and W1, W2, W3 (derived from water sample). *V. cholerae* O1 El Tor N16961, classical strain 569B O139 strain MO10 and clinical isolate O1 El Tor Ogawa IDH02365 of year 2009 were a kind gift from Dr. T. Ramamurthy, National Institute of Cholera and Enteric Diseases (NICED), Kolkata, India, The study was approved by the Institutional Biosafety Committee (IBSC) of Indian Institute of Advanced Research and Review Committee on Genetic Manipulation (RCGM) governed by guidelines laid down by Department of Biotechnology, Govt. of India. The study was also approved by the Institutional Ethical Clearance Committee of NICED. All surgery was performed under sodium pentobarbital anesthesia, and all efforts were made to minimize suffering. No specific permissions were required for collection of samples from this location as it was not situated in any private land, national park or a protected area of land. The field study did not involve any endangered or protected species.

### Pulsed Field Gel Electrophoresis (PFGE) and Random Amplification of Polymorphic DNA (RAPD) analysis

PFGE was carried out as described earlier [26]. For gel electrophoresis, 1% Bio-Rad pulsed field certified agarose gel (Bio-Rad Laboratories, Richmond, CA) was made in 0.5X TBE and run in CHEF MAPPER (Bio-Rad Laboratories) using autoalgorithm mode (molecular weight range: 100 K-350 K and a run time of 19 h). The gel was stained with 0.05 mg/ml ethidium bromide for 30 min and destained with sterile water for 1 h. RAPD was carried out using 1281 and 1283 primers by the PCR method described previously [21,27].

### PCR

Genomic DNA extraction was performed as described previously [28]. PCR assays were carried out to check the presence of the regulatory genes and virulence genes using appropriate primers as listed in Table 1 and genomic DNA as templates [29]. Each PCR involved an initial denaturation at 94°C for 4 min, followed by 30 amplification cycles each consisting of an initial denaturation at 94°C for 1 min followed by annealing and extension steps. Final polymerisation was carried out at 72°C for 10 min. For annealing and extension steps, conditions for each PCR varied depending on the Tm of the primer pair used as mentioned in Table 1. The PCR reactions were performed using PTC-225 DNA engine Tetrad (MJ Research Inc., Waltham, MA) and Taq polymerase (Bangalore Genei Pvt. Ltd., Bangalore, India).

**Table 1:** Sequences of the primers and annealing conditions in the PCRs used for the detection of virulence and regulatory genes.

**S. No**	**Primers**	**Product length (bp**)	**Primer Sequence 5’3’**	**PCR conditions**	**Reference**
**1**	ctxA-F ctxA-R	302	CTCAGACGGGATTTGTTAGGCACG TCTATCTCTGTAGCCCCTATTACG	55 °C 1 min 30 s 72 °C 1 min 30 s	**[30**]
**2**	ctxB-F ctxB-R	460	GGTTGCTTCTCATCATCGAACCAC GATACACATAATAGAATTAAGGATG	55 °C 1 min 72 °C 1 min	[31]
**3**	tcpI-F tcpI-R	862	TAGCCTTAGTTCTCAGCAGGCA GGCAATAGTGTCGAGCTCGTTA	60 °C 1 min 30 s 72 °C 1 min	**[32**]
**4**	Face Race	284	GCTTATGATGGACACCCTTTA GTTTAACGCTCGCAGGGCAAA	55 °C 1 min 30 s 72 °C 1 min 30 s	**[7**]
**5**	Fzot Rzot	243	CACTGTTGGTGATGAGCGTTATCG TTTCACTTCTACCCACAGCGCTTG	55 °C 1 min 30 s 72 °C 1 min 30 s	**[7**]
**6**	hlyA-F hlyA-R1	265	CAATCGTTGCGCAATCGCG TTGACCTTCAGCATCACT	51 °C 1 min 30 s 72 °C 1 min 30 s	**This study**
**7**	tcpA(classical)-F tcpA (El Tor) R1 tcpA (classical) R	466 466	CACGATAAGAAAACCGGTCAAGAG GATCAGCGACAGCAGCGAAA GATCTGCAAGTGCTACTGCGC	61.5 °C 1 min 72 °C 1 min	**This study**
**8**	tox R-F tox R-R	901	TTACTACTCACACACTTTGATGGCATCGTT TTAATGTTCGGATTAGGACACAACTCAAAAG	55 °C 1 min 30 s 72 °C 1 min 30 s	**[21**]
**9**	ompW-F ompW-R	586	CACCAAGAAGGTGACTTTATTGTG GAACTTATAACCACCCGCG	55 °C 1 min 30 s 72 °C 1 min 30 s	**[30**]
**10**	rtxC-F rtxC-R	265	CGACGAAGATCATTGACGAC CATCGTCGTTATGTGGTTGC	55 °C 1 min 72 °C 1 min	**[33**]
**11**	ompU-R ompU-F	655	CCAAAGCGGTGACAAAGC TTCCATGCGGTAAGAAGC	59 °C 1 min 72 °C 2 min	**[34**]
**12**	Hap F Hap R	700	GTGAACAACACGCTGGAGAA CGTTGATATCCACCAAAGG	48 °C 1 min 72 °C 1 min	**[14**]
**13**	Prt V F PrtV R	864	CATACTGAGATGCTCTACGAT TTTCACCATGTTCGGGCGTGA	50 °C 45 s 72 °C 1 min	**[14**]
**14**	**VC1649F** **VC1649R**	**842**	**GGTGGTAGTTATCTTGGTGG** **GTCACAACTCGCTCCTGAA**	**50 °C 45 s** **72 °C 1 min**	**[14**]

### Antibiotic susceptibility assay

*V. cholerae* isolates were tested for their susceptibility to ampicillin (10 µg), chloramphenicol (30 µg), co-trimoxazole (1.25 µg trimethoprim/23.75 μg sulfamethoxazole), ciprofloxacin (5 µg), gentamicin (10 µg), streptomycin (10 µg), sulfisoxazole (300 µg), trimethoprim (5 µg), tetracycline (30 µg), neomycin (30 µg), nalidixic acid (30 µg), norfloxacin (10 µg), kanamycin (30 µg) and polymyxin B (300 units) by the disk diffusion method using commercial disks (HiMedia, Mumbai, India) in accordance with the criteria recommended by Clinical and Laboratory Standards Institute (CLSI) standards [35]. When no interpretive criteria for *V. cholerae* were available based on CLSI guidelines, breakpoints for enterobacteriaceae were applied. *E. coli* ATCC 25922 was used for quality control. Experiments were performed in triplicate.

### Determination of hemolytic, hemagglutinin and protease activities

The hemolytic activity was quantitated by adding equal amount of protein from each culture supernatant to 100 µl of 10% rabbit RBC and phosphate-buffered saline (PBS) in a final volume of 1 ml. The mixtures were incubated at 37°C for 30 min and centrifuged at 2000 rpm for 5 min. Absorbance of the supernatants was measured spectrophotometrically at 540 nm. 1X PBS and triton X-100 were used as negative and positive controls respectively. The values were expressed in terms of percentage of hemolysis where the absorbance in triton X-100 control was considered 100%. For hemagglutinin activity, equal amount of protein from each culture supernatant was added in wells of a microtitre plate and 25 µl of 10% rabbit RBC were added and final volume was made up to 250 µl using PBS. The reaction was incubated at room temperature for 2 h. Lectin from the seed of the plant *Sorghum* *bicolor* (kind courtesy Dr. D. D. Singh, Department of Bioinformatics, Indian Institute of Advanced Research, Gandhinagar, India) and PBS were used as positive and negative controls respectively in this assay. Determination of protease activity was carried out as per the published protocol [36]. For determining the protease activity, equal amount of protein from the culture supernatant of each isolate was added to the wells of an agar plate containing 1.5% skim milk as a substrate and the plates were incubated at 37°C for 8 h. The halo around each well was measured after overlaying the plates with 10% trichloroacetic acid solution to achieve zone clarity. Zones with diameters of 6-8 mm were classified as low protease activity (+), 8-10 mm as intermediate activity (++) and with 10-12 mm as high activity (+++). The effect of temperature (75°C for 15 min) was also studied on the activities of these protein samples. All the assays described above were carried out in triplicates and the results were presented as average of the values obtained.

### Cytotoxicity analysis

Sterile cell free culture supernatants and cell lysates were examined for their cytotoxic activity with some modifications in the protocol already published [37]. *V. cholerae* colonies were inoculated in LB broth and incubated overnight at 37°C. Culture supernatants were obtained by centrifugation of these cultures at 5,000 rpm for 10 min. These were filter-sterilized using 0.22 µm filter units (Nalgene, Rochester, NY, USA) and saved in prechilled sterile vials. The cell pellets were washed, resuspended in PBS and subjected to sonication for 6 min (30 s on and 30 s off) in ice. After sonication, the cell lysates were centrifuged at 20,000 rpm for 5 min at 4°C, filter sterilized with 0.22 µm filter units and saved in prechilled sterile vials. Cell lysates and culture supernatants obtained from DH5α cells and PBS were taken as negative controls. Fractions from *V. cholerae* O1 El Tor N16961 were also included as controls. HT29 cells were grown in 25 cm^2^ tissue culture flasks (Corning, NY, USA) using DMEM supplemented with 10% fetal bovine serum and 1X PSN antibiotic mixture (Invitrogen, CA, USA) at 37°C in a humidified 5% CO_2_ incubator. These cells were plated at a density of 1.25 x 10^5^ cells/well in 24-well plates and incubated in CO_2_ incubator for 24 h. Subsequently, the medium was replaced with fresh medium and cells were incubated with 50 µl of culture supernatants or cell lysates serially diluted with PBS. Aliquots of each test dilution were added in triplicate to the assay plate. After 24 h, the morphological changes and cytotoxic effects of these bacterial extracts on HT29 cells were observed using an inverted phase contrast microscope (Olympus, Japan). For each well, minimum of 2-3 fields were observed and cytotoxic activity was classified as low (+), medium (++) or high (+++). The number of viable and dead cells was determined by trypan blue staining. The experiment was independently performed three times.

### SDS-PAGE analysis and protein determination

Sodium dodecyl sulfate-polyacrylamide gel electrophoresis (SDS-PAGE) was performed using the method of Laemmli [38]. Amount of protein in the culture supernatants was determined using Bradford reagent (Bio-Rad Laboratories) as per the manufacturer’s protocol and Bovine Serum Albumin was used as the standard.

### Rabbit ileal loop assay

Rabbit ileal loop experiments were conducted as described by De and Chatterjee [18]. New Zealand White male rabbits (1.5 to 2 kg) were fasted for 48 h prior to surgery and fed only water ad libitum. Rabbits were anesthetized by intramuscular administration of ketamine (35 mg/kg of body weight) and xylazine (5 mg/kg). A laparotomy was performed, and the ileum was washed and ligated into discrete loops of approximately 10 cm. Each loop was inoculated with 10^8^ CFU of challenge strain (N16961, IDH02365, W3 and Z2) in PBS, PBS was used as negative control. The intestine was returned to the peritoneum and the animals were sutured and returned to their cages. After 18 h, rabbits were sacrificed by intravenous injection of Pentobarbital (150 mg/kg), and the loops were excised. Fluid volume and loop length were measured, and secretion was recorded as milliliters per centimeter. The assay was performed three times.

## Results

### Isolation and identification of *Vibrio cholerae*

Nine environmental isolates were obtained from pond water after alkaline peptone water enrichment followed by selection on TCBS agar. The presumptive *V. cholerae* colonies were further confirmed for their authenticity using standard biochemical tests. These were found to be oxidase positive, indole positive, urease negative, citrate negative and gave positive result in mannitol motility test, typical of *V. cholerae*. Serotyping tests with O1 polyvalent antiserum and O139 monoclonal antibody showed that these isolates did not agglutinate with either of these antibodies. Hence, these isolates were inferred to belong to non-O1/non-O139 serogroups.

### Genetic relatedness between *V. cholerae* isolates

**Figure 1:**
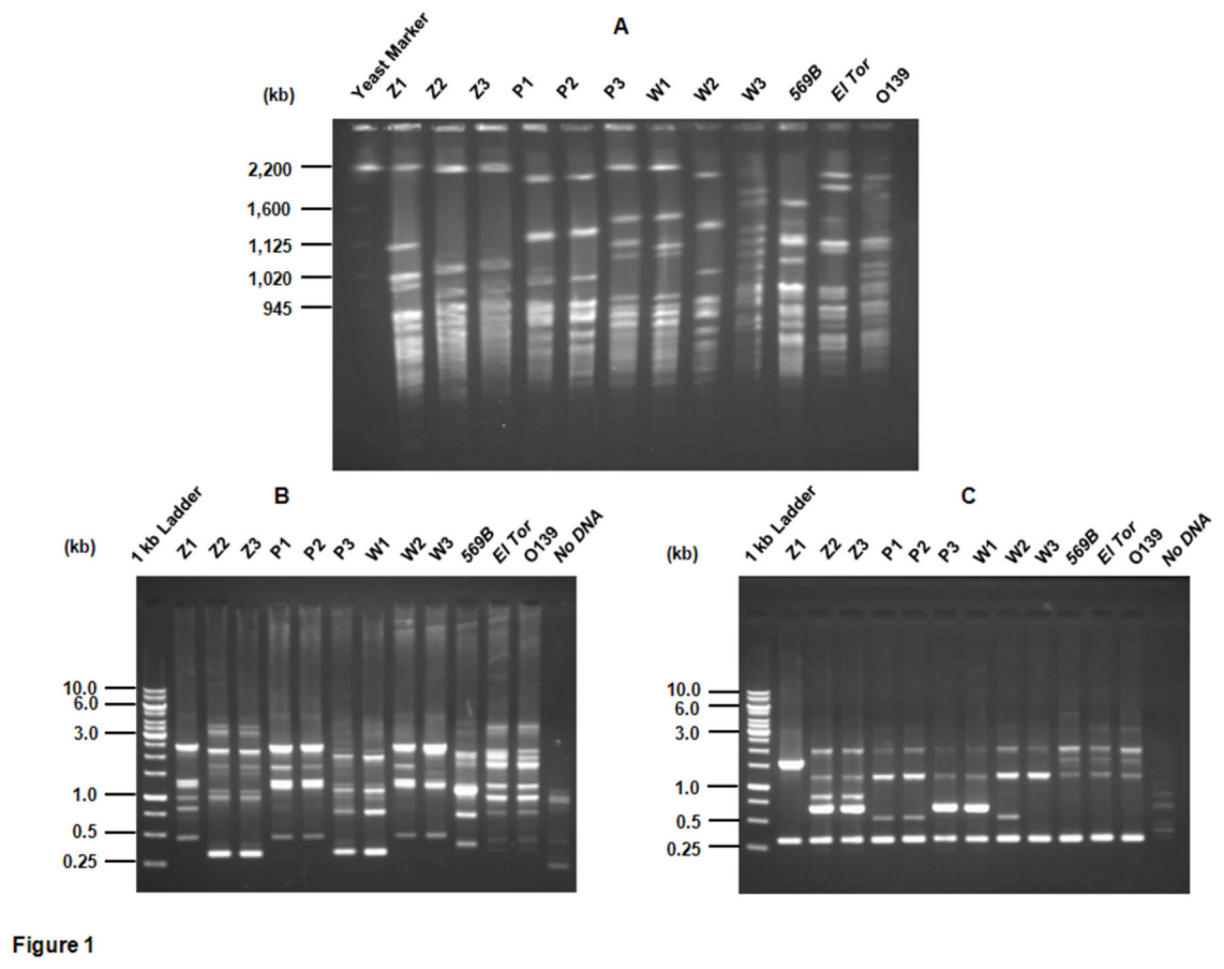
Genetic relatedness of nine environmental isolates of *V. cholerae* from Gujarat. PFGE (1%) analysis of *Sfi*I-digested genomic DNA (A) Yeast Marker: CHEF DNA size standards (Bio-Rad) from *S. cerevisiae*. Positions of the marker bands have been indicated on the left. Agarose gel (1%) analysis of RAPD using 1281 primer (B) and 1283 primer (C). In B and C, 1 kb DNA ladder (Fermentas) with fragment sizes is indicated on the left side. For all the three panels, the identity of the sample used has been indicated at the top of each lane.

As all the isolates were collected from the same place, it was of interest to find out if these isolates shared the same clonal origin. The *Sfi*I-digests of total genomic DNA of nine isolates were subjected to PFGE analysis. Five different pulsotypes were observed among these isolates (Figure 1A). Z2 and Z3 belonged to the same group, P1, P2 and W2 shared the same pulsotype whereas P3 and W1 showed the same pattern. Z1 and W3 each showed a different pulsotype which was also distinct from rest of the patterns. The PFGE patterns for all the environmental samples were also different from that of *V. cholerae* O1 classical (569B), El Tor N16961 and O139 serogroups (Figure 1A). The results of clonality using PFGE were corroborated by RAPD PCR fingerprinting analysis using 1281 and 1283 primers which also showed the same five groups (Figure 1B and 1C). These results clearly indicated heterogeneity among these environmental isolates.

### Detection of virulence genes by PCR

PCR experiments were carried out with published primers to detect the presence of different genes related to identification/virulence/toxicity of these *V. cholerae* isolates. The genes were: *toxR* (toxin regulatory gene), *ompW* (encoding the outer membrane protein specific for *V. cholerae*), *ompU* (encoding a porin in the outer membrane), *tcpA* (encoding toxin-coregulated pilus for classical/El Tor), *tcpI* (encoding toxin-coregulated pilus regulator), *rtxC* (encoding repeat toxin), *hlyA* (encoding El Tor-like hemolysin)*, hapA* (encoding hemagglutinin protease), *prtV* (encoding *V. cholerae* protease), *VC1649* (encoding *V. cholerae* protease corresponding to ORF 1649), *ctxA* (encoding cholera toxin A subunit), *ctxB* (encoding cholera toxin B subunit)*, zot* (encoding zonula occludens toxin) and *ace* (encoding accessory cholera enterotoxin). Results are summarized in Table 2 and revealed that all the strains contained the specific amplicons for *toxR*, *ompW*, *rtxC, hlyA*, *hapA* and *prtV*. Some strains contained *ompU* and *VC1649* and all the environmental strains lacked the toxigenic genes like *ctxA, ctxB, tcpA, tcpI, zot* and *ace*. Sequences for the three protease genes *hapA*, *prtV* and *VC1649* for some of the isolates were assembled and submitted to GenBank (**JQ013426,JQ013427,JQ013428,JQ013429**).

**Table 2:** PCR Analysis of environmental isolates for the presence of various genes.

**Isolate**	***toxR***	***ompW***	***ompU***	***tcpA(ElTor***)	***tcpA(class***)	***tcpI***	***rtxC***	***hlyA***	***ctxA***	***ctxB***	***zot***	***ace***	***hap***	***PrtV***	***VC1649***
**Z1**	**+**	**+**	**-**	**-**	**-**	**-**	**+**	**+**	**-**	**-**	**-**	**-**	**+**	**+**	**-**
**Z2**	**+**	**+**	**+**	**-**	**-**	**-**	**+**	**+**	**-**	**-**	**-**	**-**	**+**	**+**	**-**
**Z3**	**+**	**+**	**+**	**-**	**-**	**-**	**+**	**+**	**-**	**-**	**-**	**-**	**+**	**+**	**-**
**P1**	**+**	**+**	**-**	**-**	**-**	**-**	**+**	**+**	**-**	**-**	**-**	**-**	**+**	**+**	**+**
**P2**	**+**	**+**	**-**	**-**	**-**	**-**	**+**	**+**	**-**	**-**	**-**	**-**	**+**	**+**	**+**
**P3**	**+**	**+**	**+**	**-**	**-**	**-**	**+**	**+**	**-**	**-**	**-**	**-**	**+**	**+**	**-**
**W1**	**+**	**+**	**+**	**-**	**-**	**-**	**+**	**+**	**-**	**-**	**-**	**-**	**+**	**+**	**-**
**W2**	**+**	**+**	**-**	**-**	**-**	**-**	**+**	**+**	**-**	**-**	**-**	**-**	**+**	**+**	**+**
**W3**	**+**	**+**	**-**	**-**	**-**	**-**	**+**	**+**	**-**	**-**	**-**	**-**	**+**	**+**	**+**
***V. cholerae* classical 569B**	**+**	**+**	**+**	**-**	**+**	**+**	**-**	**+**	**+**	**+**	**+**	**+**	**+**	**+**	**+**
***V. cholerae* O1 ElTor N16961**	**+**	**+**	**+**	**+**	**-**	**+**	**+**	**+**	**+**	**+**	**+**	**+**	**+**	**+**	**+**
***V. cholerae* O139 M010**	**+**	**+**	**+**	**+**	**-**	**+**	**+**	**+**	**+**	**+**	**+**	**+**	**+**	**+**	**+**

### Antimicrobial susceptibility

The isolates were tested for antimicrobial susceptibility to fourteen antibiotics. No discrimination was made between intermediate and complete resistance and both were considered as complete resistance. All the strains showed resistance to polymyxin B and neomycin. Except W3 and P1, all the other isolates were resistant to kanamycin. In addition to polymyxin B, neomycin and kanamycin, Z2, Z3, P3 and W1 exhibited resistance to ampicillin also whereas P2 additionally showed resistance to streptomycin.

### Hemolytic activity

The hemolytic assay was carried out to quantitate the production of β- hemolysins in these isolates. The sterile culture supernatants of the isolates Z1 and W3 showed complete hemolysis (100%) of rabbit RBC whereas the culture supernatants of remaining isolates showed only 5 to 11% hemolysis (Table 3).

**Table 3:** Activities associated with the environmental isolates.

**Strains**	**Hemolytic activity (%**)*	**Hemagglutinin activity**	**Cytotoxic activity**	**Protease activity**
**Z1**	100.0	**+**	**++**	**++**
**Z2**	11.0	**+++**	**+++**	**+++**
**Z3**	7.3	**+++**	**+++**	**+++**
**P1**	8.3	**++**	**+++**	**+++**
**P2**	8.0	**+++**	**+++**	**+++**
**P3**	6.3	**++**	**+++**	**+++**
**W1**	7.3	**+++**	**+++**	**+++**
**W2**	5.3	**+++**	**+++**	**+++**
**W3**	100.0	**-**	**++**	**+**
***V. cholerae* O1 ElTor N16961**	10.6	**-**	**+**	**+**
***E. coli* DH5 α**	5.3	**-**	**+**	**+**

* The absorbance obtained from sample with triton X-100 was considered 100%.

### Hemagglutination activity

Agglutination assay was carried out with 1% rabbit RBC to detect the secretion of hemagglutinin by these environmental isolates. The degree of hemagglutination varied within these isolates (Table 3). While the culture supernatants of the isolates Z2, Z3, P2, W1 and W2 showed strong agglutination, P1 and P3 showed moderate reaction Z1 exhibited weak hemagglutination whereas in W3 and *V. cholerae* O1 El Tor N16961, no hemagglutination was observed.

### Cytotoxic activity

**Figure 2:**
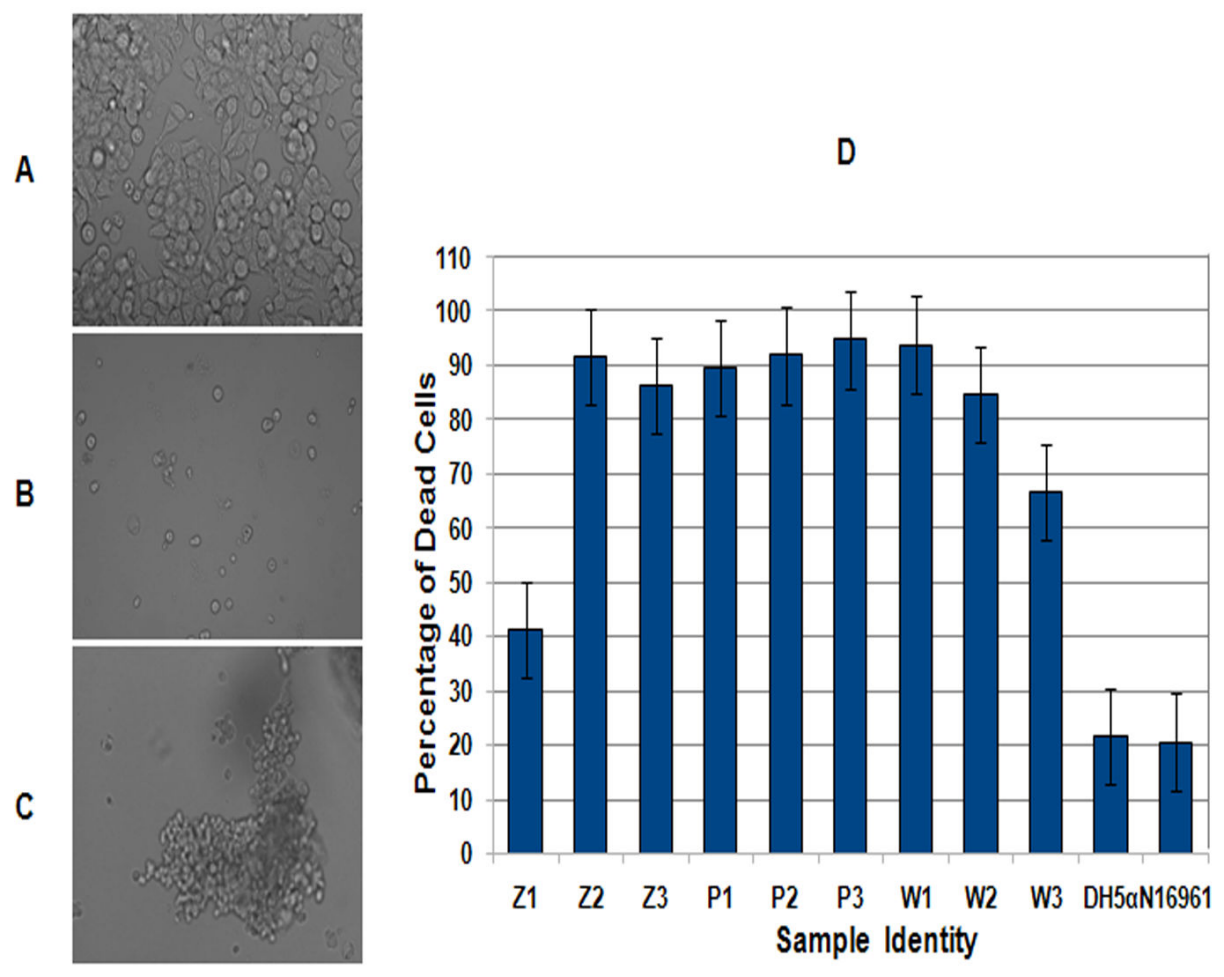
Cytotoxic effect of environmental isolates on HT29 cells. HT29 cells not treated with supernatant from any of the isolates (A); Cell rounding in HT29 cells treated with the supernatant from P1(B); Clumping of HT29 cells treated with the supernatant from Z2 (C); Bar diagram showing cytotoxic effect of culture supernatants from environmental isolates and the controls *V. cholerae* N16961 and *E. coli* DH5α in terms of percentage of dead cell (D). The experiment was independently performed three times. The error bars indicate standard deviation.

The effect of cell-free culture supernatants and cell lysates from these isolates was tested on HT29 cells, a human colon cell line. When observed under microscope, HT29 cells treated with most of the supernatants exhibited morphological features ranging from cell rounding to cell clumping (Figure 2A, B and C). However, as compared to the cell lysates, the supernatants from the isolates Z2, Z3, P1, P2, P3, W1 and W2 induced massive cell death indicating the presence of a potent extracellular cytotoxic factor in their supernatants (Table 3 and Figure 2D). Severity of the cytotoxicity was apparent in Z2 where the neat as well as the dilutions up to 100X of the culture supernatant showed excessive clumping (Figure 2C). Z1 and W3 showed moderate cytotoxicity. Interestingly, the epidemic strain *V. cholerae* O1 El Tor N16961 used as a control in this assay showed lower cytotoxicity as compared to the environmental isolates (Table 3).

### SDS-PAGE analysis of the culture supernatants

**Figure 3:**
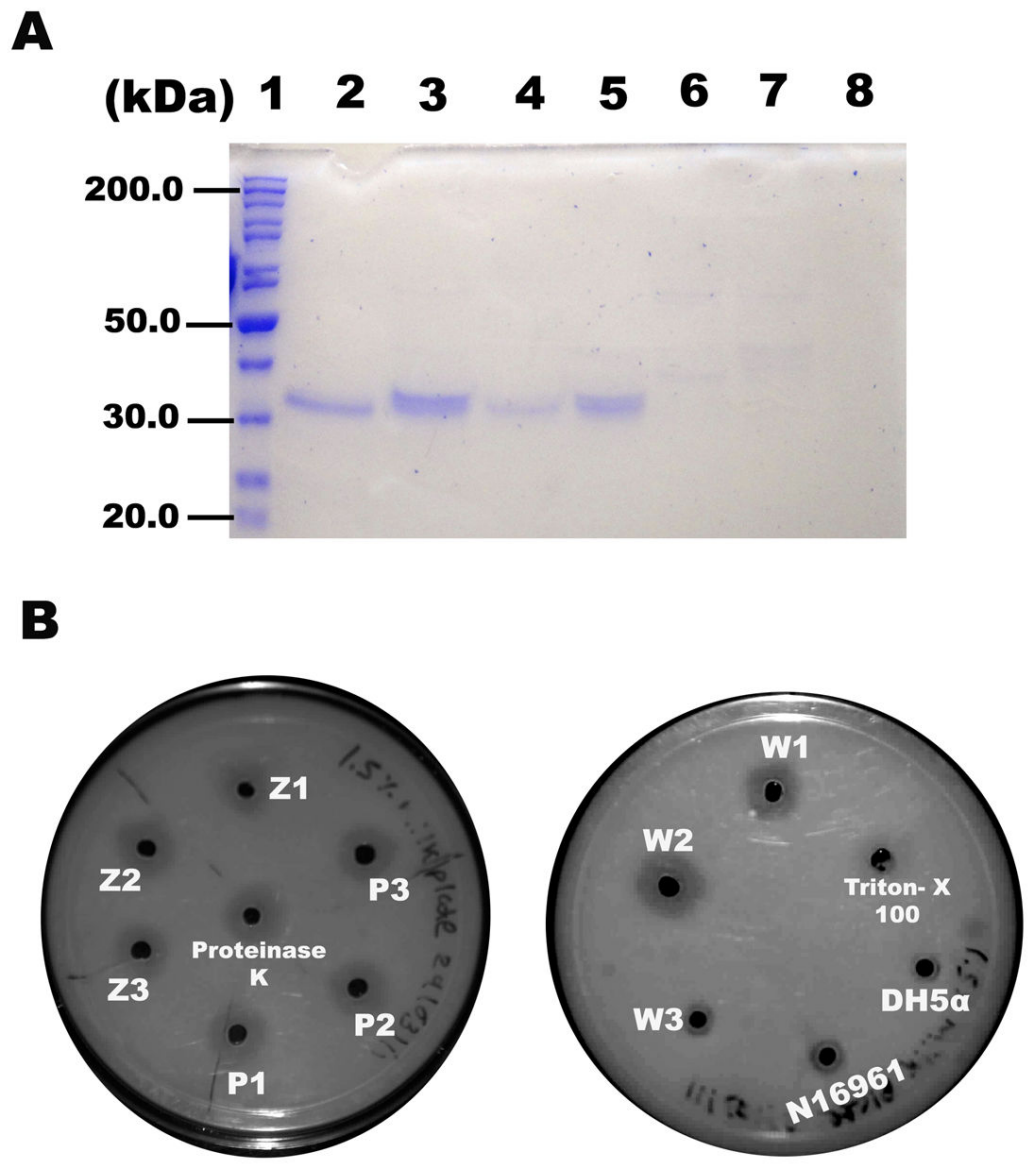
Analysis of protease activity of the environmental isolates. SDS PAGE (10%) analysis of culture supernatants (A). Lane 1: Protein markers (Pageruler, Fermentas) with molecular mass in kDa indicated on the left; Lanes 2 to 8, culture supernatants of Z2, Z3, P2, W1, W3, *V. cholerae* N16961 and *E. coli* DH5α, respectively. Protease activity of culture supernatants on 1.5% Skim milk agar plate (B). Sample identity has been indicated near each well.

Equal amount of proteins from the cell-free culture supernatants from the environmental and the control strains were electrophoresed on a protein gel under reducing and denaturing conditions (Figure 3A). The coomassie blue-stained gel showed a prominent protein band doublet slightly above 30 kDa position in the supernatants from all of the environmental isolates except in Z1, W3 and the two control strains *V. cholerae* O1 El Tor N16961 and DH5α. The mobility of this doublet corresponded to a very well documented protease, HA protease or a hemagglutinin protease [11,12].

### Protein identification by MS peptide mapping and sequence analysis

The Coomassie blue stained protein band at 30 kDa position was excised from the gel and analysed on Bruker Ultraflex III MALDI instrument. Trypsin digestion and peptide mass fingerprinting followed by Protein identification using Mascot software confirmed the protein to be a hemagglutinin protease. Peptides that matched the *V. cholerae* hemagglutinin protease (gi 446704828) were K.SGWNVR.K, R.AFYLLANKSGWNVR.K, R.AMTFGDGYTR.F, R.YFDQPSRDGR.S, R.GNVDWIVGADIFK.S, R.VHYGNNYENAFWDGR.A, R.DMSGGINEAFSDIAGEAAEYFMR.G, R.DGRSIDHASQYYSGIDVHYSSGVFNR.A, K.VVFDMYQQWLNTSPLTFQLTMR.V and R. FYPLVDINVSAHEVSHGFTEQNSGLVYR.D.

### Protease activity

When tested on an agar plate containing 1.5% skim milk as a substrate, all the strains showed high protease activity except W3 (Table 3 and Figure 3B). The protease activity was abolished on treating the supernatants at 75°C for 15 min. In addition, careful analysis revealed a correlation between the cytotoxic activities and protease activities for all the isolates tested (Table 3).

### Enterotoxic activity

**Figure 4:**
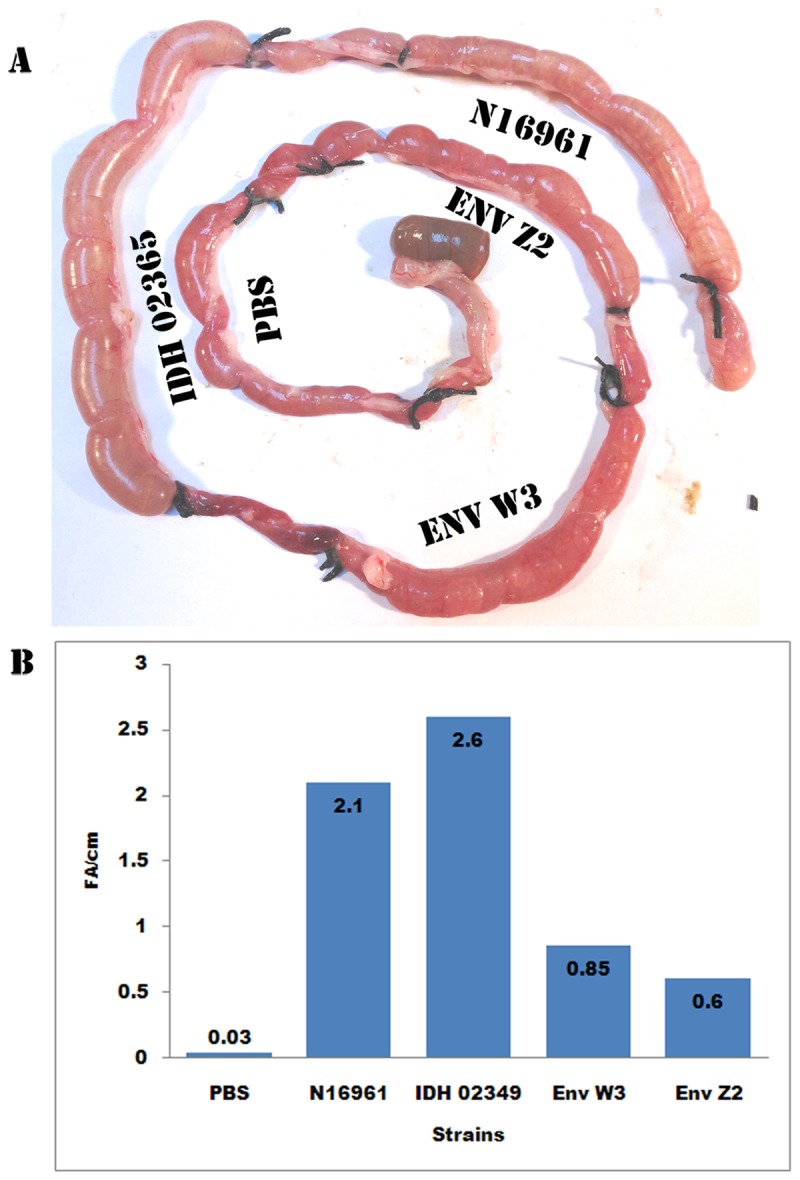
Rabbit ileal loop assay to assess entrotoxigenic activity. Pictorial view of rabbit ileal loop of different clinical and environmental strains (A). Analysis of fluid accumulation of different *V. cholerae* strains (B). Rabbit ileal loops were inoculated with 10^8^ CFU of each strain N16961 (O1 El tor, clinical), IDH02365(O1 El tor, clinical), Z2 (non-O1/non-O139, Environmental) and W3(non-O1/non-O139, Environmental) in PBS and incubated for 18 h. Results are expressed as fluid accumulation (FA) (in milliliters) per loop length (in centimeters). Shown are means ± standard deviations; *n* = 3.

Two of the isolates Z2 (representing high cytotoxicity) and W3 (representing high hemolytic activity) were tested in rabbit ileal loop assays to assess their fluid accumulation-causing potential. PBS, *V. cholerae* O1 El Tor N16961 and a recent clinical isolate of *V. cholerae* O1 El Tor Ogawa IDH02365 were taken as controls in this assay. Results revealed that W3 caused fluid accumulation in the range of 0.72-0.93 whereas Z2 yielded the range of 0.6-0.72 ml/cm. The control strains N16961 and IDH02365 showed fluid accumulation in the range of 2.0-2.2 and 2.6-2.7 ml/cm respectively (Figure 4).

## Discussion

In the present study, nine environmental *V. cholerae* isolates were investigated to determine their toxin profiles and predict whether these isolates could act as reservoirs of virulence/infection. These environmental *V. cholerae* non-O1/non-O139 biotype El Tor isolates were found to be negative for *tcp*, *ctx*, *ace* and *zot* genes but carried the genes for ToxR, El Tor-like hemolysin, repeat toxin, hemagglutinin protease and another *V. cholerae* protease PrtV. It is also possible that the environmental strains might have carried variant forms of the genes that have not been detected in this study using PCR as has been observed earlier for the *tcpA* gene [2,39].

In most of the cases *V. cholerae* non-O1/non-O139 strains lack cholera toxin but have been implicated to cause moderate to severe gastroenteritis in humans [11]. Apart from cholera toxin, several other extracellular and intracellular proteins have been found to be responsible for virulence/pathogenicity/cytotoxicity in *V. cholerae* O1, O139 and non-O1/non-O139 strains. These include Shiga-like toxin, hemolysins, hemagglutinin protease to name a few [16,40,41]. Proteases dismantle the host tissue barriers and help in the invasion and pathogenicity process. For example, the processed 32 kDa form of hemagglutinin protease (HAP), plays a crucial role in the pathogenicity of non-O1/non-O139 strains [11,12]. HAP is responsible for nicking of A-subunit of cholera toxin and in liberation of N-terminal region of haemolysin from its precursor [40]. Directly or indirectly, HAP plays a main role in pathogenesis of *V. cholerae* that are devoid of cholera toxin. Similarly, PrtV protease has been implicated in virulence of *Vibrios* in *Caenorhabditis elegans* model system. This protease gives the *Vibrios* an added advantage to survive in aquatic niches [42].

In this study, varying degrees of cytotoxicity to HT29 cells were observed with culture supernatants from the environmental isolates indicating that the cytotoxicity trait was associated with the supernatants that showed remarkably higher cytotoxicity than the cell lysates. This clearly hinted that the factor(s) responsible for the observed cytotoxicity was a secreted protein(s)/extracellular protein(s). In comparison to the epidemic strain *V. cholerae* N16961, these isolates were more cytotoxic. It has been previously reported that the cytotoxic effect of non-O1/non-O139 *V. cholerae* is probably related to the production of hemolysin [43]. However, in this study although all the isolates were positive for the *hlyA* gene corresponding to El Tor-like hemolysin, the degree of hemolysis varied among the isolates, with Z1 and W3 showing 100% hemolysis. No correlation was observed between hemolytic activity and cytotoxic potential of the isolates indicating that hemolytic potential may not be the only factor contributing to the cytotoxicity. Further analysis of hemagglutinin and protease activities showed that these were higher in all the isolates except Z1 and W3. Additionally, the cytotoxicity appeared to be a manifestation of both hemagglutinin and protease activities. The isolates Z1 and W3 were found to behave differently from rest of the seven isolates in all the biochemical assays. This finding also corroborated our earlier observation in PFGE and RAPD analysis where these two isolates showed distinct patterns than rest of the isolates. Though they showed strongest hemolytic potential, the other three activities (hemagglutinin, cytotoxic and protease activities) were weakest in these two isolates. SDS-PAGE analysis of the culture supernatants from these isolates also confirmed the above observation. While the seven isolates contained a protein doublet at 32 kDa corresponding to the molecular size of processed HAP, the Z1 and W3 supernatants were devoid of this band. Presence of a doublet also depicted the processing of a protease to produce its mature active form as has been documented earlier [11,36]. Though all the isolates carried the genes for both hemolysin and HAP, the protein expression and activities seemed to vary among these isolates. Most interestingly, the hemolytic, protease, hemagglutinin and cytotoxic potentials of environmental isolates were much higher as compared to the epidemic diarrhoeal strain *V. cholerae* O1 El Tor N16961 (Table 3). Though these isolates lacked cholera toxin, the synergistic effect of other virulence factors rendered higher toxicity to these isolates. Several environmental factors and cell signals may be involved in triggering the higher expression of these virulence factors in environmental isolates.

To assess *in vivo* activity of these environmental isolates, the ligated rabbit ileal loop assay was performed. These isolates had demonstrated higher activities as compared to N16961 epidemic strain in *in vitro* assays described in table 3. Therefore, for *in vivo* evaluation of their toxic potential, another fresh clinical isolate IDH02365 was included to ascertain that the lower activities obtained with N16961 were not due to its multiple passages in the laboratory. Previous studies have correlated the enterotoxigenicity of *V. cholerae* strains with their hemolytic potential [9,20,43]. To confirm the relationship and evaluate the enterotoxigenicity, two representative isolates were chosen for the rabbit ileal loop assay. One of these isolates (Z2) had low hemolytic activity and high cytotoxicity whereas the other (W3) had high hemolytic activity and lower cytotoxicity. The fluid accumulation ratios were found to be almost similar in both these isolates. Therefore, in the present study, the enterotoxigenic potential could not be attributed to hemolytic activity or any other activities described in Table 3. It is also possible that some other mechanisms yet undiscovered could play a role in fluid accumulation apart from the factors tested in this study and other studies done so far [9,20,43]. In addition, *in vivo* toxicity of these two environmental isolates was found to be comparable to the clinical strains *V. cholerae* N16961 and IDH02365. Similar results have also been obtained in another study where the strains of *V. cholerae* O1 lacking the CTX virulence cassette were able to evoke mild to moderate fluid accumulation in the rabbit ileal loop assay [44]. *In vivo* colonization model such as the suckling mouse might help to characterize these environmental isolates better and this aspect needs to be investigated further.

There have been attempts to unravel the mechanisms of pathogenicity in environmental isolates from various regions of India where cholera is truly endemic and epidemic [1,16,34,45,46]. This study presents various extracellular and intracellular putative mechanisms of toxicity associated with environmental isolates from Gujarat and suggests that these mechanisms could lead to inflammatory and hemorrhagic responses in synergy with each other. Bacterial species encountering a broad spectrum of environment during the course of their life cycle are likely to develop complex regulatory systems and stress adaptation mechanisms to best survive in each environment. By acquisition of virulence genes, toxigenic strains have evolved from the environmental non-pathogenic strains [47,48]. Certain environmental and host factors may trigger the conversion of these environmental isolates into more toxic forms that could be the progenitors for an outbreak. A recent study has also demonstrated the contribution of non-O1/non-O139 *V. cholerae* in causing the Haiti outbreak and serving as a reservoir for genomic and pathogenicity islands [49]. The present study is an attempt to understand the synergy among various toxic/ virulence factors as a manifestation of expression of pathogenicity of environmental isolates. To the best of our knowledge, this is a first comprehensive effort to address the contribution of different proteins in the hyper-toxicity of the environmental isolates from Gujarat.
